# Raltegravir Inclusion Decreases CD4 T-Cells Intra-Cellular Viral Load and Increases CD4 and CD28 Positive T-Cells in Selected HIV Patients

**DOI:** 10.3390/cells11020208

**Published:** 2022-01-08

**Authors:** Gaurav Kumar, Jacqueline Cottalorda-Dufayard, Rodolphe Garraffo, Francine De Salvador-Guillouët, Eric Cua, Pierre-Marie Roger

**Affiliations:** 1 Unité 576, Centre Hospitalier Universitaire de Nice, Institut National de la Sante et de la Recherche Medicale, Universite de Nice-Sophia-Antipolis, 06200 Nice, France; pierre-marie.roger@chu-guadeloupe.fr; 2 Arthritis and Clinical Immunology, Oklahoma Medical Research Foundation, Oklahoma City, OK 73104, USA; 3 Virologie, Hopital l’Archet 2, Centre Hospitalier Universitaire de Nice, Universite de Nice-Sophia-Antipolis, 06200 Nice, France; Dufayard.J@chu-nice.fr; 4 Pharmacologie, Hopital Pasteur, Centre Hospitalier Universitaire de Nice, Universite de Nice-Sophia-Antipolis, 06200 Nice, France; Rodolphe.Garraffo@unice.fr; 5 Infectiologie, Hopital l’Archet 1, Centre Hospitalier Universitaire de Nice, Universite de Nice-Sophia-Antipolis, 06200 Nice, France; guillouet.f@chu-nice.fr (F.D.S.-G.); cua.e@chu-nice.fr (E.C.); 6 Service Des Maladies Infectieuses et Tropicales, Centre Hospitalier Universitaire de Pointe-à-Pitre, 97159 Pointe-à-Pitre, France

**Keywords:** HIV infection, raltegravir, CD4 T-cells, pharmacokinetics, viral load

## Abstract

Raltegravir (RLT) prevents the integration of HIV DNA in the nucleus, but published studies remain controversial, suggesting that it does not decrease proviral DNA. However, there are only a few studies focused on virus-targeted cells. We aimed our study on the impact of RLT inclusion on total intra-cellular viral DNA (TID) in cellular subsets and immune effects in patients with newly acquired undetectable plasmatic viral load (UVL). Six patients having UVL using an antiretroviral combination for 6 months and CD4 T-cells > 350/mL and <500/mL were selected to receive RLT for 3 months from M0 to M3. Patients had 7 sequential viro-immunological determinations from M-1 to M5. Immune phenotypes were determined by flow cytometry and TID quantification was performed using PCR assay on purified cells. TID (median values) at the initiation of RLT in CD4 T-cells was 117 copies/millions of cells, decreased to 27.5 on M3, and remained thereafter permanently under the cut-off (<10 copies/millions of cells) in 4 out of 6 patients. This was associated with an increase of CD4 and CD4 + CD28+ T-cells and a decrease of HLA-DR expression and apoptosis of CD4 T-cells. RLT inclusion led to decreases in the viral load along with positive immune reconstitution, mainly for CD4 T-cells in HIV patients.

## 1. Introduction

The morbidity and mortality related to Human Immunodeficiency Virus (HIV) infection have dramatically decreased in countries where antiretroviral combinations are available [1]. A better clinical outcome is usually seen in HIV patients that receive antiretroviral therapy (ART) in the early phase of viral infection following HIV diagnosis [2]. Most of the time, undetectable viral load is reached within a few months after the beginning of ART, which is associated initially with a rapid rise in CD4+ T-cell followed by a subsequently slower increase over the years of treatment [3]. However, a major limitation of ART lies in the fact that it is still incapable of completely restoring the normal immune response which could suppress viral replication after ART has been stopped [4].

To date, preventing the establishment of the viral reservoir and its replication has been a major limitation, even after several years of ART [5]. CD4+ T-cells and monocyte-lineage are the most important targets for HIV-1. These cells are the main cellular reservoirs in which the virus can persist and survive for long periods within the body [6,7,8]. These two different T-cell types may help in continuous replication of the virus despite efficient ART [9,10]. Viruses infect activated CD4 T-cells that are transitioning back to the resting memory state. The stably integrated provirus remains transcriptionally inactive in the long-lived memory T-cells thereby evading host immune response and ART [11]. HIV viruses infect circulating monocytes which further differentiate into macrophages. These infected macrophages contribute significantly to viremia during ART, as the viral replication in CD4 T-cells is inhibited [12]. Cellular reservoirs and tissue sanctuaries allow continuous low-level viral transcription and contain integrated proviral DNA despite continuous ART [13,14]. These data necessitate the search for an efficient ART with potential to eliminate the cellular provirus for a sustained therapeutic benefit.

T-cell activation has been reported to be a predictor of HIV disease progression and can even predict ART treatment failure [13]. HIV viruses specifically target the T-cells by exploiting the costimulatory molecules CD28 and CTLA-4, resulting in the excessive loss of CD4 T-cells with an increase in CD8 T-cells and HIV infection. This is reported in part through down-regulation of CD28 expression and overexpression of CD152 [14,15,16,17]. Expression of these two abnormal membrane-bound proteins is associated with enhanced T-cell apoptosis, a hallmark of HIV infection [18,19]. Additionally, downregulation of CD80 and CD86 markers on T-cells occurs during HIV infection, which greatly impedes T-cell function in eliminating viral replication [20,21]. Given the active role of the interaction between CD28/CD152-CD80/CD86 for antigen presentation and development of an effective immune response, it might be possible that the HIV virus evades this pathway for its replicative benefits [22]. Excessive cellular activation and alterations of numerous membrane proteins such as costimulatory molecules, including CD28 and CD152, are still detected [15,16,17,23,24,25]. These results imply persistent perturbations of homeostasis at the cellular level despite efficient ART.

Raltegravir (RLT) is a potent HIV-1 integrase inhibitor, restraining the HIV-DNA integration into the host genome [26,27,28]. Although RLT has been reported to rapidly improve the CD4 and CD8 T-cell count, the associated immunological recovery has not been reported so far [29,30]. Therefore, we investigated the hypothesis that the inclusion of RLT might decrease the intracellular reservoir, and as a result, improve the T-cell homeostasis by modulating the costimulatory pathway.

Our study was aimed to measure the impact of RLT on the intra-cellular viral reservoir, in both lymphocytes and monocytes, and to measure the impact of this treatment on T-cell homeostasis, with emphasis on costimulatory molecules. We also sought to determine the RLT concentrations in purified cells, as there are only very few reports on pharmacokinetic measurements of RLT inside the HIV-targeted immune cells (i.e., CD4 T-cells and monocytes).

## 2. Materials and Methods

### 2.1. Study Subjects

Six HIV-infected patients attending the Infectious Disease Department of Nice University Hospital, France, were recruited for this prospective study (5 males, 1 female, age range 31–65 years old). This study was realized in accordance with the ethical committee of our institution and the patients gave their informed consent in compliance with our institutional review board.

The inclusion criteria were to be infected with HIV-1, presenting with an undetectable viral load for at least 6 months and a CD4 T-cell count between 350 and 500 CD4 T-cells/mm^3^, using ART based on two nucleoside reverse transcriptase inhibitor (NRTI) and one protease inhibitor (PI) for no more than one year.

RLT was added to the ART combination of the patient for 3 months (400 mg twice daily). Two samples were taken before adding RLT, and two after stopping it, allowing the study of 7 consecutive time points i.e., day −30 and day 0 (before RLT intake); day 30, day 60, and day 90 (during RLT intake); and day 120 and day 150 after RLT withdrawal.

The exclusion criteria included previous use of an integrase inhibitor, patients with active opportunistic infection and/or cancer as well as those patients who benefited from recombinant IL-2, interferon-α, steroids, or other medications known to modify immune cell reactivity.

### 2.2. Sample Processing, Cell Separation, and Cell Culture

Blood samples were drawn from patients in tubes containing acid citrate dextrose (ACD) in the Infectiologie unit of the hospital and brought to the lab immediately for processing. Whole blood was centrifuged to obtain the plasma. Peripheral blood mononuclear cells (PBMCs) were isolated from the blood using Ficoll-Paque PLUS (Stemcell Technologies, Vancouver, BC, Canada) density gradient centrifugation. For analysis of phenotypic and functional markers, PBMCs were used immediately after their isolation and counting. To observe the alteration in proliferation and apoptosis, T-cells were stimulated overnight with CD3 and CD28 at 37 °C with 5% CO_2_ in RPMI 1640 (Gibco, Grand Island, NY, USA) supplemented with 10% of fetal calf serum (Thermo Fisher Scientific, Hampton, NH, USA). For separation of CD4 T-cells and monocytes from PBMC, we used positive (monocytes) and negative (CD4 T-cells) magnetic separation kits (Stemcell Technologies, Vancouver, BC, Canada), and the purity of the cells was verified by flow cytometry. After counting, the cells were stored as pellets along with plasma and PBMC at −80 °C for viral load and pharmacological analysis. All the purification steps were performed at 4 °C in order to prevent drug loss from the cells.

### 2.3. Cell Stimulation with CD3/CD28

Cell culture was performed in coated wells with anti-CD3 mAbs and anti-CD28 mAbs added in solution (clone 28-2, 10 μg/mL final dilution). Wells were coated as follows: 500 µL of medium containing CD3 mAbs (clone X3, 10 µg/mL final dilution), incubated for 2 h at 37 °C; wells were then washed 3 times with the medium alone. Clone X3 and 28-2 are produced in our laboratory.

### 2.4. Monoclonal Antibodies

The following conjugated Anti-human monoclonal antibodies (mAbs) were purchased from BD Pharmingen, USA: CD3 PE-Cy7, CD4 FITC, CD4 PerCP-Cy5.5, CD4 APC, CD8 PerCP-Cy5.5, CD14 PerCP-Cy5.5, CD14 PE, CD80 FITC, CD86 PE, CD152 PE, HLA-DR APC, Ki67 PE, Annexin V FITC, and Propidium Iodide. CD28 FITC and CD45 Pacific blue were purchased from eBioscience, USA.

### 2.5. Multiparameter Flow Cytometry

For the extracellular labeling of the cells, one million PBMCs were taken in each tube, and antibodies were added to it. Thereafter, cells were incubated at 4 °C in the dark for 20–30 min, washed twice with PBS, and analyzed using flow cytometry (FACS CANTO II, BD Biosciences, NJ, USA) within 24 h of labeling. For intracellular labeling (Ki67), the cells were firstly labeled with extracellular antibodies, washed in PBS, fixed, and permeabilized. Lastly, cells were stained with mAbs along with an isotype-matched control mAbs (Becton Dickinson, San Jose, CA, USA). We used cytofluorometric techniques with a six-color labeling pattern (FITC/PE/PerCP-Cy5.5/PE-Cy7/APC/Pacific blue).

### 2.6. Viral Load Analysis

Total HIV intra-cellular DNA (TID) of PBMC, CD4, monocytes, and plasmatic viral load (pVL) was analyzed using a commercially available PCR assay. Total DNA of each purified population was isolated by using QIAmp DNA blood mini kit (Qiagen, Hilden, Germany) and then the total TID was PCR amplified using HIV DNA cell kit (Biocentric, Bandol, France), and, finally, the amplified viral DNA was quantified to know the exact copies of viral DNA inside PBMC, CD4, monocytes, and plasma.

### 2.7. Total Plasma and Cellular Concentration

Raltegravir and anti-protease cellular concentrations in plasma, PBMC, CD4, and monocytes were measured by HPLC coupled to tandem mass spectrometry (LC-MS/MS; AB Sciex, MA, USA). A 200 µL volume of extracting solution containing RLT was added to each isolated cell population and plasma. The lysates of each cell population were then vortexed, sonicated for 30 min, and finally centrifuged at 14,000 rpm for 10 min at 20 °C. A 200 µL volume of supernatant was introduced into a microvial and a 20 µL volume was injected into the LC-MS/MS apparatus for drug quantification. C_cell_ was expressed in ng/mL according to the cell count for all the cell populations.

### 2.8. Statistical Method

Biological parameters were compared from baseline to D90 by using the Wilcoxon signed-rank test, with an alpha risk (two-sided) of 0.05. Statview^®^F-4.5 software (SAS Institute Inc., Cary, NC, USA) was used for all analyses. Paired *t*-test was performed using Prism 8 (GraphPad, San Diego, CA, USA). Only significant differences are indicated. The results are expressed as medians (range).

## 3. Results

Six HIV patients who fulfilled the inclusion criteria after extensive screening were enrolled for the study. The main characteristics of the HIV patients including age, sex, duration of HIV, ARV duration and regimen, and their CD4 T-cell counts are described in Table 1.

### 3.1. Plasmatic and Cellular Viral Load

**Figure 1:**
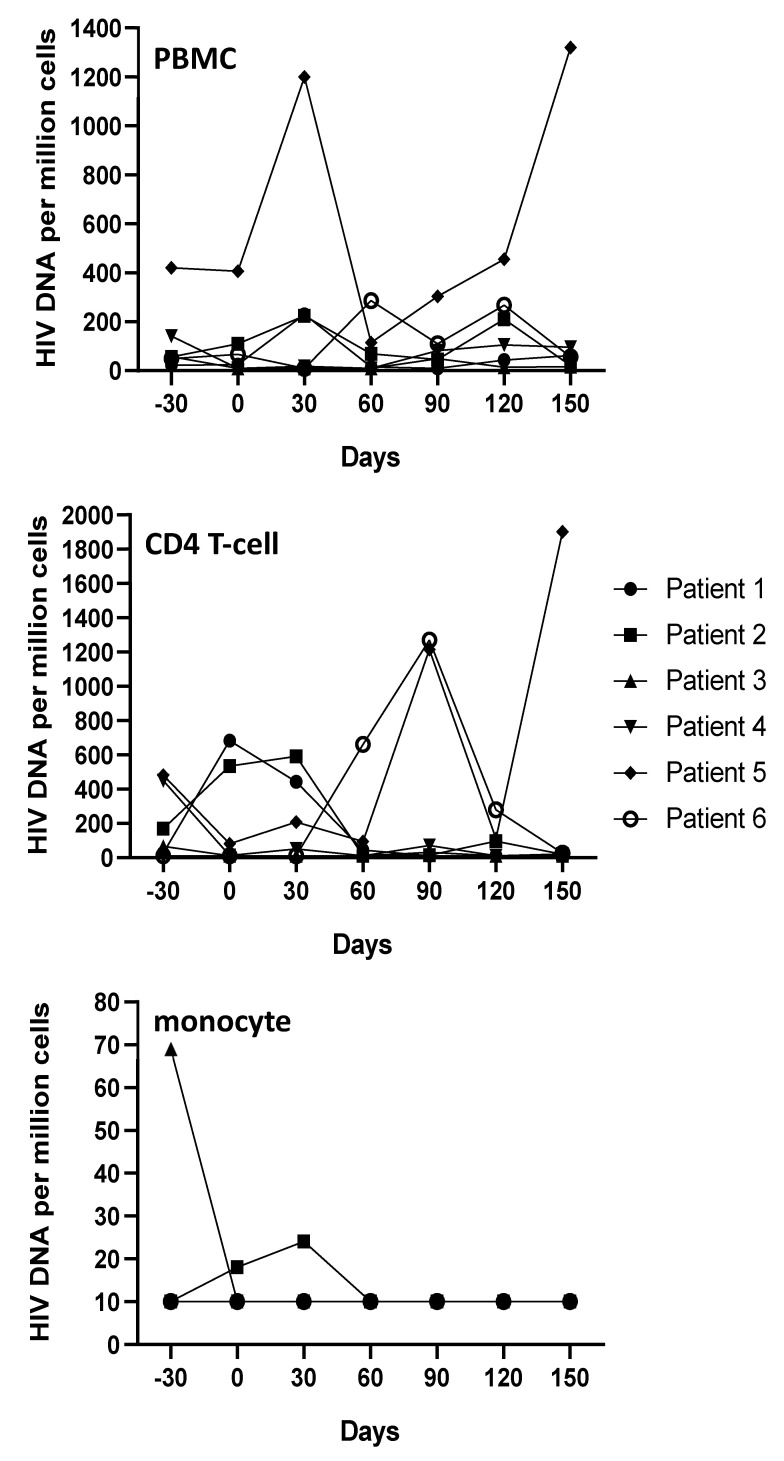
Kinetics of intracellular viral DNA over 7 months of the study protocol. Raltegravir was associated with the current antiretroviral combination from Day 0 to Day 90 (*X*-axis) with quantification of intracellular HIV DNA per million cells (*Y*-axis), in peripheral blood mononuclear cells (PBMC), purified CD4 T-cells, and monocytes. A higher quantity of HIV DNA was detected in CD4 T-cells, while monocyte concentrations appeared low to undetectable. Variations of intracellular HIV DNA in PBMC were unpredictable, but it was decreasing in CD4 T-cell subsets, except in patients 5 and 6, as well as in monocytes in which no HIV DNA was detected over Day 60.

The plasmatic viral load remained undetectable during the course of RLT treatment in all patients. In PBMC, the TID median value was 57 (23–421) on day −30 which reduced to 43 (10–115) on day 60. On day 90, all 6 patients showed a decreasing trend of PBMC TID as compared to day −30, but a favorable outcome was seen only in 2 out of 6 patients after RLT administration, throughout the course of the study (Figure 1). We observed a decreasing trend for CD4 T-cell TID, the median value at the beginning being 117 copies/millions of cells (11–480), which reached its minimum load of 27.5 (10–660) on day 60, and 30 (11–1214) on day 90. The CD4 T-cell TID remained permanently under the cut-off (<10 copies/millions of cells) in 4 out of 6 patients after day 60 (Figure 1). For monocytes, the total TID was most of the time under the lower limit of detection, except for 3 time-points in 2 different patients before RLT inclusion (Figure 1).

### 3.2. T-Cell Count, Activation and Costimulatory Molecules

**Figure 2:**
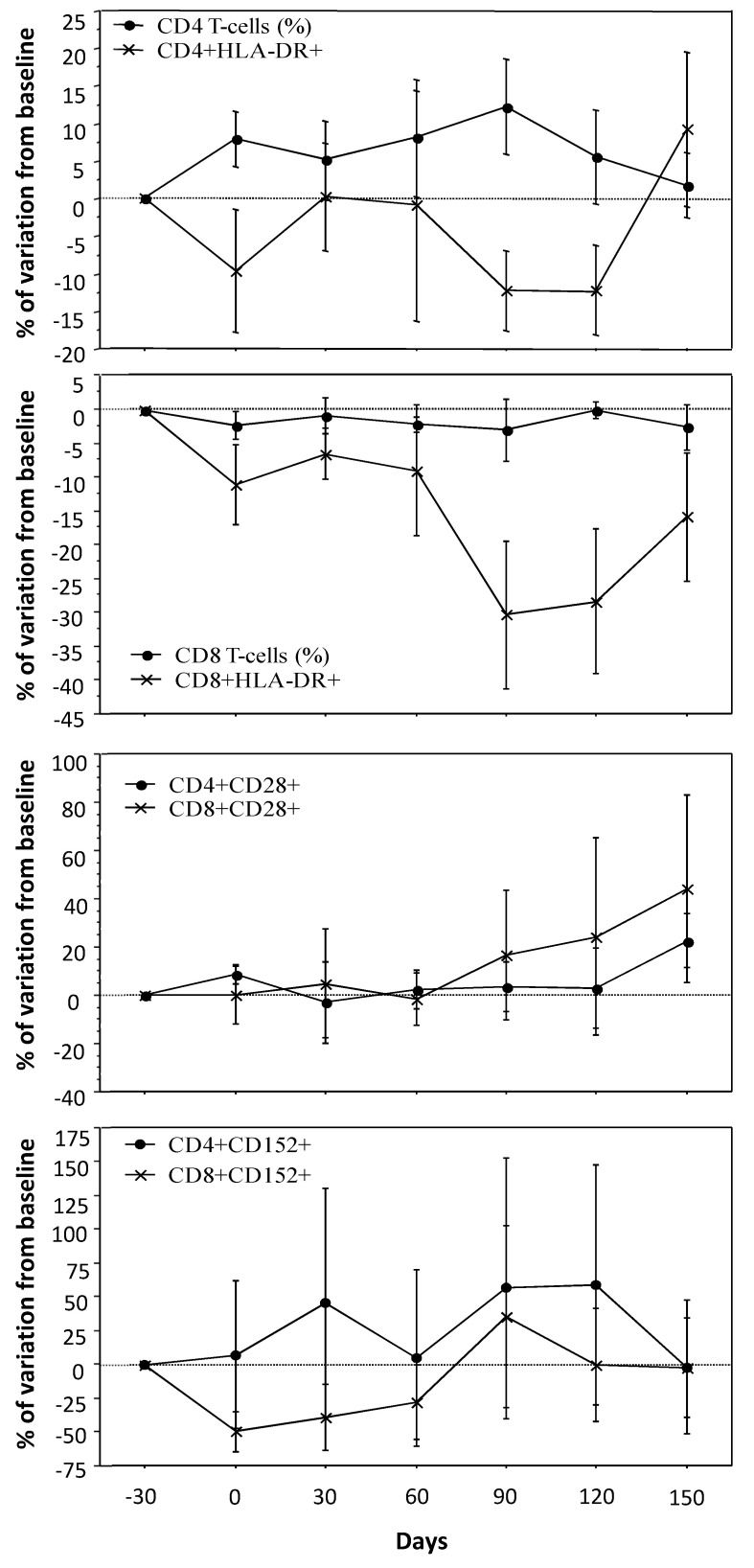
Kinetics of T-cell recovery, activation markers, and costimulatory molecules. T-cell subsets were differently affected during raltegravir inclusion, with a slight increase in CD4 T-cells, contrasting with a trend towards lower CD8 T-cell count. In both cases, HLA-DR positive T-cells were decreasing with rough up-regulation by the end of raltegravir inclusion. Regarding costimulatory molecules, only CD28 positive T-cells showed a regular slight increase, while CD152 expression was not clearly affected.

The median values for CD4 T-cells at baseline was 451 cells/mm^3^ (351–476), which reached 534 cells/mm^3^ (433–557) on day 90, *p* = 0.018. By the end of the study, the median CD4 T-cell was 516/mm^3^ (367–762) (Figure 2). The impact of RLT inclusion on absolute CD4 T-cell count is associated with a trend towards a higher percentage from baseline to day 90. At day 150, a paired analysis showed a significant increase in CD4 T-cell percentage as compared to baseline, *p* = 0.0425 (Figure S1). Accordingly, Figure 2 suggests that this increase of CD4 T-cells was coupled with a slight decrease of activation as measured through HLA-DR expression. On day 90, the percentage of activated CD4 T-cells was significantly decreased as compared to baseline, *p* = 0.0484 (Figure S1).

Absolute CD8 T-cell counts were 677 cells/mm^3^ (552–949) at baseline, 686 (458–905) on day 90 and 689 (502–954) on day 150. Despite these stable CD8 T-cell counts, these T-cell subsets also showed a decrease of activated phenotype (Figure 2). On day 90, the paired analysis showed a significant decrease in activated CD8 T-cells percentage, *p* = 0.0386 (Figure S1).

Among costimulatory molecules, only CD28 expression showed a coherent increase, particularly on CD8 T-cells (Figure 2 and Figure S1). CD152 positive cell kinetics followed a bumpy curve which ruled out any interpretation (Figure 2 and Figure S1). Finally, the expression of CD80 and CD86 did not show significant variation over the course of the study (data not shown).

### 3.3. Proliferation Versus Apoptosis

**Figure 3:**
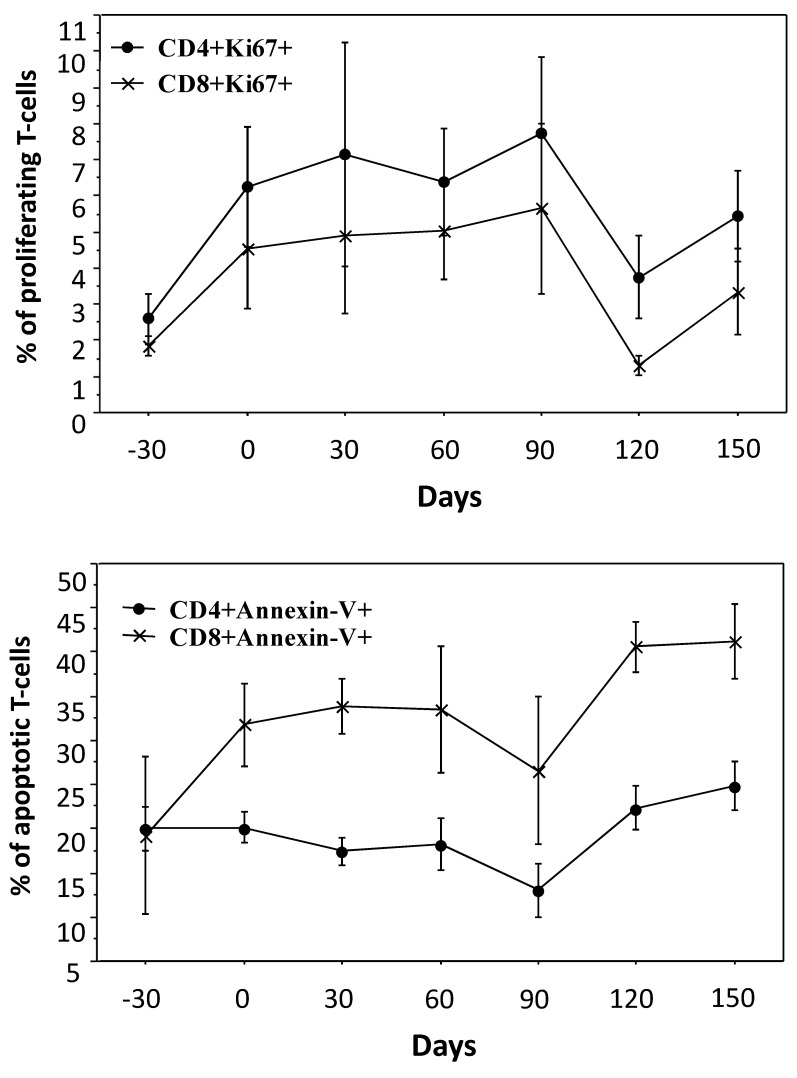
Kinetics of T-cell proliferation and T-cell apoptosis. Raltegravir inclusion had clearly 2 different effects on T-cell subsets. For CD4 T-cells, RLT allowed both a slight increase in proliferation, measured using the Ki67 marker, associated with a trend towards lower apoptosis in CD4 T-cells, measured using the Annexin-V marker. Regarding the CD8 T-cell subset, both markers were increased. It should be noted that raltegravir interruption induced bumpy modifications in the percentage of T-cell subsets.

We measured the proliferation of T-cells by the expression of the Ki67 marker. For all the 6 patients studied, the value for the proliferation of CD4 T-cells at baseline was 2.1% (0.8–5.0), which reached 7.2% (1.5–14.2) on day 90 and 4.9% on day 150 (1.2–9.4). For CD8 T-cells, the values were 1.6% (0.9–2.7), 3.2% (0.5–13.2), and 2.6% (1.2–9.4) respectively (Figure 3).

The apoptotic T-cells were recognized as positive for Annexin-V and negative for propidium iodide. Kinetics of T-cell apoptosis was clearly different between T-cell subsets, being decreased for CD4 T-cells and up-regulated for CD8 T-cells. The median value of apoptotic CD4 T-cells at baseline was 20% (13–27), which gradually went down to 12.5% (2–23) on day 90, being 25% (17–33%) on day 150. For CD8 T-cells, respective values were 14% (1–53), 20% (3–56), and 43% (21–50) (Figure 3). Our data clearly shows a positive outcome under RLT, suggesting the biological effect of inclusion, and worsening or returning to baseline after withdrawal.

### 3.4. Monocyte Phenotype

We used CD14 antibodies for the detection of monocytes. It should be considered that monocytes spontaneously expressed HLA-DR, and that cellular activation leads to an up-regulation of membrane expression of this molecule which is detected on the cytofluorometric experiment by the increase of mean intensity fluorescence (MIF). Respective MIF values at baseline, day 90 and day 150 showed a trend towards a down-regulation of HLA-DR expression, being 32116 (15222–39935), 21875 (3174–38665), and 26188 (18563–48282) respectively (*p* = 0.08). We also tested monocytes for the expression of costimulatory molecule ligands, i.e., B7 molecules, namely CD80 and CD86, which were not altered by RLT inclusion (data not shown).

### 3.5. Intracellular Concentrations of Raltegravir

**Figure 4:**
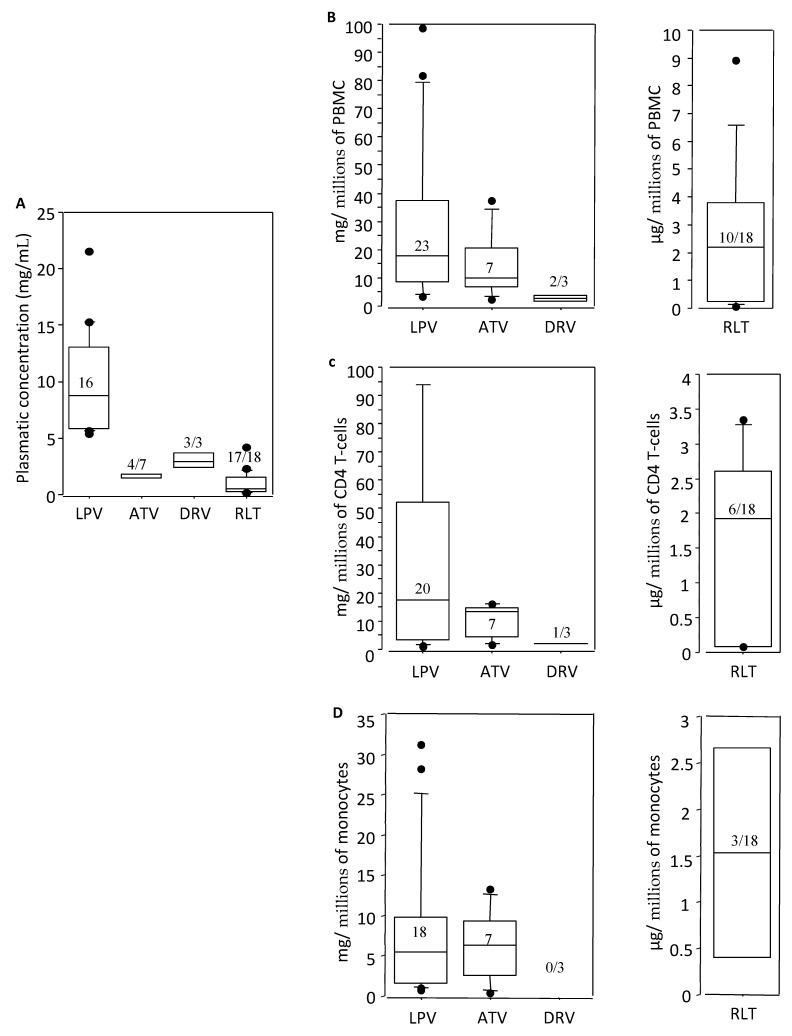
Intracellular drug concentrations in Plasma, PBMC, CD4 T-cells, and monocytes. The cumulative pharmacological data of the 4 drugs analyzed at all the time points. As lopinavir determinations were used to test our methodology, the number of samples is over the expected value. The cellular drug concentration was measured per million cells. The histogram represents only the detected positive values during all time points. Of note, darunavir drug concentration was detected only at (**A**) 3 time-points in plasma, (**B**) 2 time-points in PBMC, (**C**) 1 in CD4 T-cells, and (**D**) remained undetectable in monocytes. Additionally, most of the time, raltegravir drug concentration was undetectable (77%). The number in the box represents the number of positive time-points/total time-points analyzed. LPV = Lopinavir, ATV = Atazanavir, DRV = Darunavir and RLT = Raltegravir. A. Plasmatic determinations. B. PBMC determinations. C. CD4 T-cell determinations D. Monocyte determinations.

We looked for the penetration of protease inhibitors and RLT in plasma, PBMC, CD4 T-cells, and monocytes. For protease inhibitors, plasma concentrations of the drugs were in the normal range. Raltegravir was detected in 17/18 time-points in plasma in all the patients (94%), the median value being 560 µg/mL (140–4200 µg/mL) (Figure 4A). In PBMC, RLT was detected in 10/18 time-points (56%) for all the patients considered, the detectable values being low (Figure 4B). RLT was in a detectable concentration in 6/18 time-points for CD4 T-cells (33%) (Figure 4C) and for monocytes in 3/18 time-points (Figure 4D).

## 4. Discussion

In patients living with HIV infection, most dying cells are not infected. It is suggested that T-cell fate is dependent on the micro-environment, including cell-cell interactions [24,31,32,33]. Additionally, numerous studies have demonstrated the relationship between the efficiency of antiretroviral treatments and CD4 T-cell recovery, the decrease of immune activation, including the downregulation of HLA-DR, CD38, Fas/CD95, and decrease of T-cell apoptosis [14,15,22,34]. However, the positive effect of additional ART treatment has not been clearly demonstrated for patients already with efficient antiretroviral combinations. The occurrence of anti-integrase offered the opportunity to test the effect of RLT inclusion on viral replication and immune restoration.

We report herein that RLT decreased total TID in CD4 T-cells for 4/6 patients, and in monocytes for 2 patients for whom TID was in the detectable range. A decrease of total TID in CD4 T-cells has already been reported when prescribed early in the disease [35]. Additionally, Vallejo et al. reported a significant effect of RLT inclusion in 9 patients who showed a decrease of infectious units per million of resting CD4 T-cells [36]. Our report suggests that the addition of RLT may also decrease total TID in monocytes. Previous studies focused on PBMC have failed to demonstrate any favorable effect of RLT intensification [37,38,39], suggesting that this non-specific cellular approach could be the reason for missing the virologic impact of this therapy.

An increase in CD4 T-cell count with the inclusion of RLT to combination ART has been already reported, but there are some discrepancies [35,36,37,38,39,40,41]. In our study, RLT inclusion was associated with a favorable effect on T-cell homeostasis: CD4 T-cells slightly increased during RLT inclusion in some patients, exhibited a decrease in HLA-DR expression, a late up-regulation of CD28, and a decrease of apoptosis. On the other hand, CD8 T-cells decreased during RLT treatment, exhibited a decrease in HLA-DR expression as well as an up-regulation of CD28. However, no clear impact was seen on CD8 T-cell apoptosis or proliferation. Such discrepancies between T-cell subset fate have been already reported, CD8 T-cells being mostly driven by HIV replication [22,34,40,41].

We also report a trend towards a lower HLA-DR expression on monocytes during RLT treatment, suggesting a down-regulation of cellular activation. As HIV in monocytes was most of the time under the limit of detection, cellular deactivation seems to be independent of the viral reservoir.

Of note, it is important to take into account the kinetics of immunological changes under ART [17,34,40,41], explaining our choice for a short duration of RLT treatment, already known to rapidly inhibit viral replication [27]. Our favorable results might be also related to our method, the patients benefiting from a recent antiretroviral combination and on/off periods of RLT treatment, avoiding non-significant results related to different immunologic backgrounds [37,38]. This has also been observed in a comparative trial to different HIV-1 subspecies [42] or previous long-term efficient ART treatment [43].

Finally, RLT intra-cellular concentrations were regularly undetectable or dramatically lower than protease inhibitor concentrations. It should be noted that plasmatic concentrations of both protease inhibitors and RLT were inside the range of expected values, in accordance with previous reports [44]. Results of studies on intra-cellular RLT concentrations are conflicting, with most studies suggesting low intra-cellular distribution and large inter-patient variability, while potential methodological pitfalls might explain these results [45,46]. Multiple factors, such as resistance to drugs by T cells and monocytes, cellular mutations, low or sub-optimal dosage, etc. have been associated with low integration of the drug. Comparatively, multiple mutations are required to confer raltegravir resistance in T cells, but a single mutation confers resistance in the monocytes [47,48,49]. Interestingly, we did not observe any significant difference of RLT concentrations in cellular subsets.

A major limitation of our study is the small number of patients and the limited availability of the sample. This is due to the strict inclusion criteria followed by extensive screening of the patients. Therefore, further studies are needed, to more precisely determine the kinetics of RLT in HIV cellular targets in a larger patient cohort.

In conclusion, in patients with a mild immune response to a recent first-line efficient antiretroviral combination, RLT inclusion decreases total intra-cellular HIV DNA in virally targeted cells despite low concentrations of intracellular drugs and is associated with an improvement of T-cells homeostasis.

## Figures and Tables

**Table 1:** Main characteristics of the 6 included patients. Nadir CD4 T-cells were observed before antiretroviral combinations, while CD4 max. indicates maximal CD4 T-cells value obtained during raltegravir inclusion. HIV follow-up indicates the duration of medical care since the diagnosis of HIV infection. ARV = antiretroviral combinations. LO = lopinavir, AT = atazanavir, DA = darunavir, FTC = emtricitabine, TDF = tenofovir.

Patient No.	Sex	Age	HIV Follow-Up (Months)	ARV Duration (Months)	Antiretroviral Regimen	CD4 Nadir	CD4 Max.
1	M	50	18	12	LO + FTC + TDF	357	675
2	M	34	17	10	LO + FTC + TDF	252	576
3	M	31	15	12	LO + FTC + TDF	221	897
4	M	38	34	7	AT + FTC + TDF	235	528
5	F	65	13	12	LO + FTC + TDF	205	672
6	M	34	26	8	DA + FTC + TDF	188	501

## Data Availability

The data presented in this study are available on request from the corresponding author.

## Supplementary Materials

The following are available online at https://www.mdpi.com/article/10.3390/cells11020208/s1, Figure S1: The median values of the percentage of CD4 T-cells, CD8 T-cells, and the expression of HLA-DR, CD28, and CD152 by CD4 and CD8 T-cells at different time points analyzed by flow cytometer. Error bars represent the range of the values.
