# Supplementary material for: Raltegravir Inclusion Decreases CD4 T-Cells Intra-Cellular Viral Load and Increases CD4 and CD28 Positive T-Cells in Selected HIV Patients
Source: Cells. 2022 Jan 8;11(2):208. doi: 10.3390/cells11020208 (PMC8773801; doi:10.3390/cells11020208)

Figure S1: The median values of the percentage of CD4 T-cells, CD8 T-cells and the expression of HLA-DR, CD28 and CD152 by CD4 and CD8 T-cells at different time points analyzed by flow cytometer. Error bars represents the range of the values.

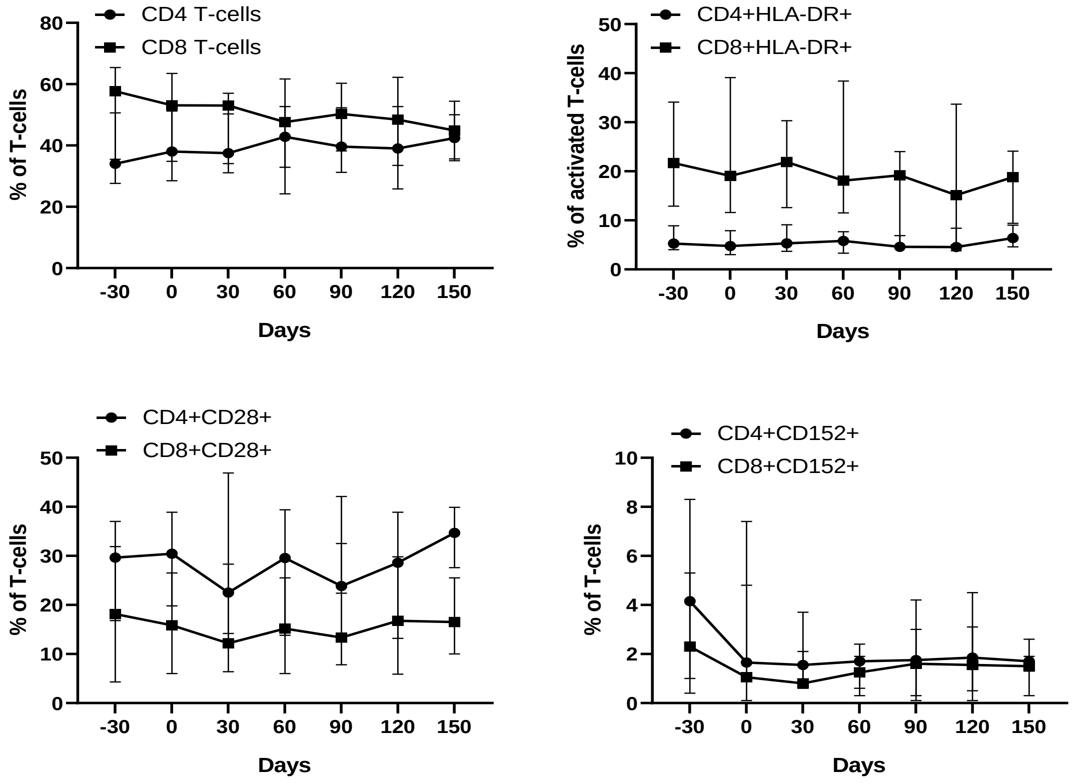

Supplement: Supplementary file 1 [file cells-11-00208-s001.zip › cells-1504239-supplementary.pdf]
